# Consecutive Application of Biogas Slurry Improved the Cumulative Nitrogen Use Efficiency by Regulating the Soil Carbon Pool

**DOI:** 10.3390/plants15010102

**Published:** 2025-12-29

**Authors:** Sheng Wu, Tingfeng Gao, Chenxue Wu, Haoqiang Yuan, Ying Liu, Jiating Liu, Lei Han, Cheng Zhang, Youhua Ma, Xia Liao

**Affiliations:** 1 Key Laboratory of Farmland Ecological Conservation and Pollution Prevention and Control in Anhui Province, College of Resources and Environment, Anhui Agricultural University, Hefei 230036, China; 2 Agricultural Technique Extension Center of Guoyang County in Anhui Province, Bozhou 233600, China; 3 College of Animal Science and Technology, Anhui Agricultural University, Hefei 230036, China

**Keywords:** biogas slurry, wheat yield, nitrogen use efficiency, lime concretion black soil, soil carbon pool

## Abstract

To identify the optimal substitution ratio of biogas slurry to chemical fertilizer, this study determined the cumulative nitrogen use efficiency (CNUE) of wheat and carbon pool in Lime concretion black soil. The following treatments were applied: control (CK), conventional chemical nitrogen fertilizer application (CN), optimized chemical fertilizer application (ON), and biogas slurry replacing 15% (ONL15%), 30% (ONL30%), and 50% (ONL50%) of fertilizer. The results indicated that CNUE was the highest in the ONL30% treatment and 67.26–80.26% higher in the ONL15%, ONL30%, and ONL50% treatments than it was the CN treatment. The soil dissolved organic carbon of 2023–2024 increased by 11.93–22.93% compared to that in the CN treatment, and the highest particulate organic carbon content was observed in ONL30% treatment. In 2024, the carbon pool management index was 22.20, 42.42, and 29.34% higher in ONL15%, ONL30%, and ONL50% treatments than it was in CN treatment, respectively. In summary, biogas slurry replacing 30% of fertilizer regulated the carbon pool in Lime concretion black soil and improved the yield, quality, and cumulative nitrogen use efficiency of wheat, which was the optimal substitution ratio of biogas slurry to chemical fertilizer in the Northern Anhui Plain of China.

## 1. Introduction

With the rapid development of large-scale livestock and poultry farming, concentrated manure discharge has become a serious ecological and environmental challenge. Anaerobic fermentation, as the main method of manure treatment used currently, produces large amounts of biogas slurry. If not effectively utilized, it can cause severe environmental pollution and resource waste [1,2]. The biogas slurry is rich in nitrogen, phosphorus, potassium, water-soluble organic carbon, and biologically active substances, making it useful as an organic liquid fertilizer. Returning it to farmland enables nutrient recycling and reduces waste pollution; thus, it has become the preferred approach for utilizing biogas slurries [3,4]. In developed countries in Europe and North America, manure application is generally regulated based on nutrient balance principles. The biogas slurry is returned to farmland through irrigation, promoting the integration of crop and livestock farming [5]. Organic matter and metabolites, such as amino acids, in the biogas slurry improve soil fertility and promote agricultural green transformation [6]. Therefore, promoting the application of biogas slurry as a fertilizer and advancing crop–livestock circular systems are of great significance for improving resource utilization efficiency [7].

As an important organic resource, biogas slurry, when applied in combination with chemical fertilizers in line with crop nutrient requirements, ensures the safe disposal of livestock waste and controls pollution and greenhouse gas emissions at the source, reducing chemical fertilizer use, enhancing soil carbon sequestration, and promoting green and sustainable agricultural development [8]. Returning biogas slurry to farmland effectively improves soil fertility and crop yield and quality. Substituting chemical fertilizers with biogas slurry can significantly increase the soil organic matter, available phosphorus and potassium contents, and the nitrate nitrogen content [9]. In garlic cultivation, a substitution ratio of 35% has been shown to be optimal, but 100% substitution has shown synergistic improvements in yield, quality, and soil fertility [10,11]. In addition, biogas slurry enhances soil microbial activity, enzyme activity, and aggregate stability, thereby promoting nutrient availability and nitrogen uptake by crops, which improves the yield and quality of grain crops [12,13,14,15,16]. The microbial populations it contains may also regulate crop metabolism and suppress plant diseases [17]. However, excessive or improper biogas slurry application may reduce the crop yield and cause issues such as soil contamination with heavy metal elements and antibiotics [18,19]. Therefore, determining an appropriate substitution ratio of biogas slurry is crucial for balancing crop production and ecological safety.

Soil organic carbon (SOC) and its components are key indicators for determining soil quality and health, directly influencing crop yield and quality [20]. Lefroy et al. proposed the carbon pool management index (CPMI), which sorts SOC into active and non-active forms, providing an effective tool for evaluating carbon pool dynamics [21]. Indicators, such as readily oxidizable organic carbon (ROC), dissolved organic carbon (DOC), and particulate organic carbon (POC), are commonly used to characterize these carbon forms [22]. The full substitution of chemical fertilizers with biogas slurry promotes carbon pool accumulation [23], and long-term livestock manure application can significantly increase the total SOC content and enhance carbon transformation and sequestration in large soil aggregates [24,25]. Xia et al. found that the substitution of 25–50% nitrogen with manure could result in an annual carbon sequestration of 268–496 kg·ha^−1^ [26]. Farmland soils in China have great potential for carbon sequestration, with an estimated annual capacity of 390 Tg, accounting for about 35% of total soil carbon sinks [27]. Biogas slurry substitution increases the total SOC and its active portion, improving CPMI, optimizing the humus composition, and increasing the proportion of humic acid, which enhances soil fertility [28,29]. Therefore, studying the partial substitution of chemical fertilizers with biogas slurry has significant practical value for promoting crop–livestock integration and the sustainable development of ecological agriculture in China.

Most studies have focused on the effects of traditional organic fertilizers and straw return on soil fertility and crop yield improvement. There is a lack of comprehensive research on the impact of substituting chemical nitrogen fertilizer with biogas slurry on wheat yield and quality and soil carbon pool properties in the region with Lime concretion black soil, particularly regarding the mechanisms underlying soil carbon pool dynamics and the optimal substitution ratio. Based on the properties of Lime concretion black soil and in response to regional needs for chemical fertilizer reduction, fertilizer efficiency improvement, and resource utilization of livestock and poultry manure, we conducted field experiments to systematically determine the combined effects of different biogas slurry substitution ratios on the soil CPMI, wheat yield, quality, and cumulative nitrogen use efficiency. The purpose of this study was to determine an appropriate application rate and biogas slurry substitution ratio for this region and to provide theoretical support and practical guidance for developing scientifically grounded manure return strategies, reducing chemical fertilizer input, and ensuring agricultural product quality and safety.

## 2. Results and Analysis

### 2.1. Effects of the Consecutive Partial Substitution of Chemical Nitrogen Fertilizer with Biogas Slurry on Wheat Yield and Its Components

**Figure 1:**
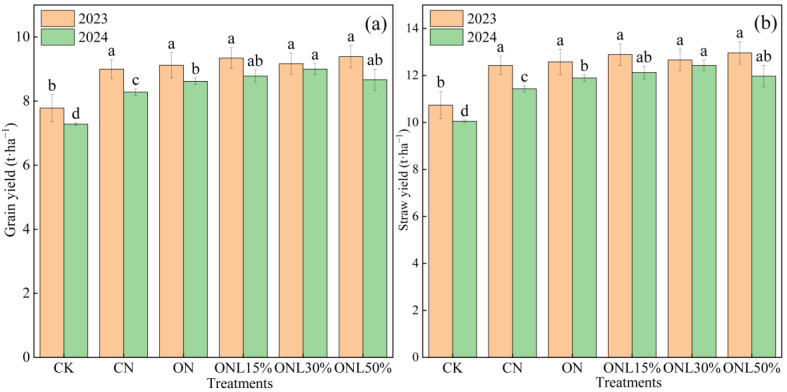
(**a**,**b**) Effects of different fertilization treatments on wheat straw and grain yield. Note: Values followed by different letters within each column are significantly different at *p* < 0.05 in the same year.

Partial substitution of chemical nitrogen fertilizer with biogas slurry had a significant effect on wheat yield and its components (Figure 1). In 2023, ONL15%, ONL30%, and ONL50% increased wheat yield by 1.90–3.76% compared to CN. In 2024, ON, ONL15%, ONL30%, and ONL50% significantly increased wheat grain and straw yield compared to CN, and ONL30% significantly increased wheat grain and straw yield compared to ON (*p* < 0.05). Compared to CN, ON treatment significantly increased 1000-grain weight in both years (Table 1), and in 2024, ONL30% significantly increased 1000-grain weight by 8.96%. Substitutions of chemical nitrogen fertilizer with biogas slurry increased wheat yield by improving the yield components, of which ONL30% increased wheat yield and farmers’ income.

### 2.2. Effects of Consecutive Partial Substitution of Chemical Nitrogen Fertilizer with Biogas Slurry on Wheat Cumulative Nitrogen Use Efficiency

**Figure 2:**
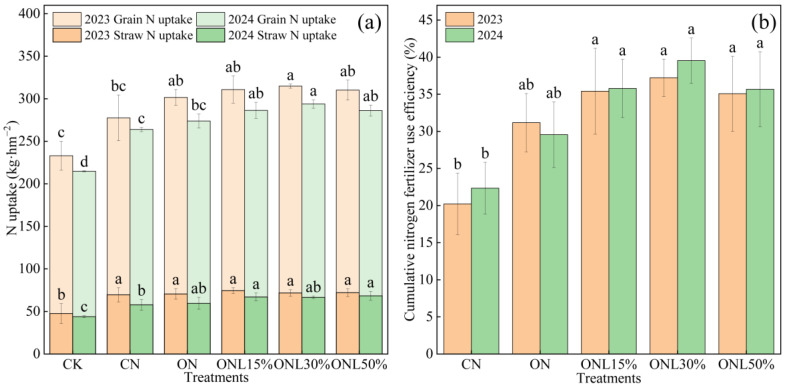
Effects of different fertilization treatments on nitrogen absorption (**a**) and cumulative nitrogen fertilizer use efficiency (**b**) of wheat. Note: Values followed by different letters within each column are significantly different at *p* < 0.05 in the same year.

Partial substitution of chemical nitrogen fertilizer with biogas slurry effectively promoted nitrogen uptake and utilization in wheat plants and significantly increased cumulative nitrogen use efficiency (Figure 2). In 2023 and 2024, ONL15%, ONL30%, and ONL50% increased wheat grain and straw nitrogen uptake by 10.04–13.57% and 8.69–11.18%, respectively, compared to CN. The cumulative nitrogen use efficiency was 35.60%, 38.37%, and 35.37% in ONL15%, ONL30%, and ONL50%, respectively, showing a significant increase of 67.26–80.26% compared to that in the CN group and an increase of 16.50–26.40% compared to that in the ON group (*p* < 0.05). Plant nitrogen uptake was further enhanced with consecutive biogas slurry application over the years.

### 2.3. Effects of Consecutive Partial Substitution of Chemical Nitrogen Fertilizer with Biogas Slurry on Wheat Quality and Heavy Metal Element Content

**Figure 3:**
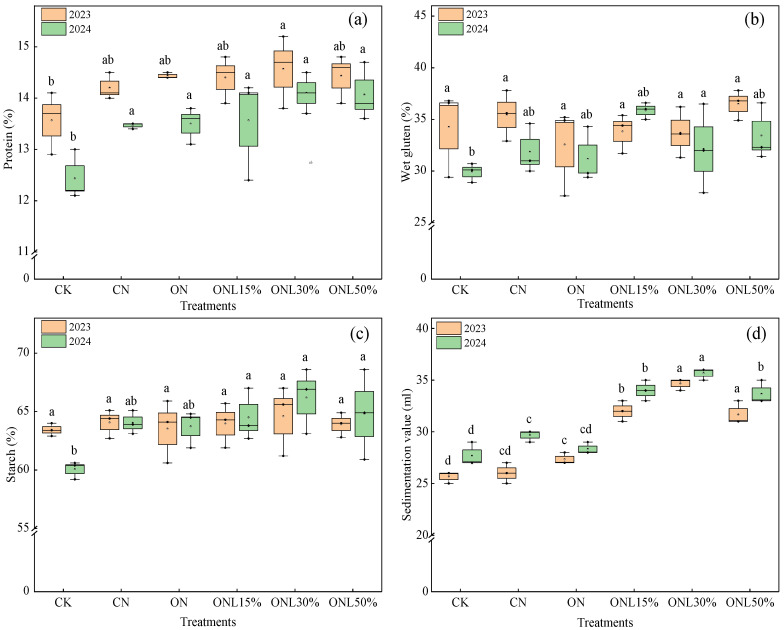
(**a**–**d**) Effects of different fertilization treatments on the quality of wheat. Note: Values followed by different letters within each column are significantly different at *p* < 0.05.

Substitution of chemical nitrogen fertilizer with biogas slurry significantly improved wheat quality (Figure 3). The data in 2023 and 2024 showed that all biogas slurry application groups showed grain protein content improvement, which was the highest in ONL30% (14.33%), followed by ONL50% (14.25%). The wet gluten content improved in all biogas slurry application groups, and the highest (35.87%) was observed in ONL15% in 2024, showing a significant 12.5% increase compared to CN. The starch content was highest in ONL30% in both years, reaching 66.4% and 66.2%, respectively. Biogas slurry application significantly increased the grain sedimentation value. In 2023, the grain sedimentation value increased by 23.08%, 33.33%, and 21.79% in ONL15%, ONL30%, and ONL50%, respectively, compared to CN; in 2024, the increases were 14.59%, 20.21%, and 13.47%, respectively. As shown in Table 2, the wheat grain heavy metal element content in all substitution groups was below the limit set by the National Food Safety Standard—Maximum Levels of Contaminants in Foods (GB 2762-2022), meeting the safety standards for consumption. Therefore, the reasonable application of biogas slurry effectively improves wheat quality and planting benefits and ensures the safety of agricultural products.

### 2.4. Effects of Consecutive Partial Substitution of Chemical Nitrogen Fertilizer with Biogas Slurry on Soil Quality

Substitution of chemical nitrogen fertilizer with biogas slurry effectively improved the soil physicochemical properties (Table 3). From 2023 to 2024, the soil pH ranged between 5.58 and 6.19 across all treatment groups and increased with a higher rate and longer duration of biogas slurry application. The soil pH in ONL50% increased by 0.40 in 2024 compared to 2023. On average, biogas slurry application decreased the soil bulk density by 4.65–6.17%. The total nitrogen was highest (1.49 g·kg^−1^) in ONL30%, with an increase of 8.33% compared to CN. The soil available phosphorus content was also increased by biogas slurry application, with increases of 4.97–9.44% and 9.98–14.63% in 2023 and 2024, respectively, compared to CN. The available potassium content was highest in ONL30%, with an increase of 5.59% compared to CN. In addition, as shown in Table 4, the heavy metal element content in all substitution group did not exceed the risk screening values specified in the Soil Environmental Quality—Risk Control Standard for Soil Contamination of Agricultural Land (Trial) (GB 15618-2018).

### 2.5. Effects of Consecutive Partial Substitution of Chemical Nitrogen Fertilizer with Biogas Slurry on Soil CPMI and the Contents of Soil Organic Carbon and Its Active Portion

Substituting chemical nitrogen fertilizer with biogas slurry effectively increased the SOC content and its active portion. As shown in Table 5, ONL30% increased SOC by 6.76% compared with CN in 2023, although the difference was not significant. In 2024, ONL15%, ONL30%, and ONL50% increased SOC by 1.48%, 7.97%, and 3.35%, respectively, compared to CN. In both years, the annual increase in soil DOC in all biogas slurry application groups ranged between 11.93 and 22.93%. The annual ROC was highest (4.84 g·kg^−1^) in ONL30% and was 4.48 g·kg^−1^ in ONL50%, showing an increase of 30.59% and 20.93%, respectively, compared to CN. The highest POC (5.08 g·kg^−1^) was observed in ONL30% in 2023, showing an increase of 14.89% compared to CN. In 2024, the highest POC (5.38 g·kg^−1^) was observed in ONL15%, with a significant increase of 25.11% (*p* < 0.05) compared to CN. In addition, between 2023 and 2024, ONL15%, ONL30%, and ONL50% significantly increased CPMI compared to CN, showing increases of 22.20–23.27%, 41.45–42.42%, and 29.34–30.94%, respectively, with ONL30% always presenting the highest CPMI. The results indicate that returning the biogas slurry to the field promotes soil active organic carbon formation and nutrient circulation, with the substitution ratio of 30% showing the optimal effect.

### 2.6. Correlation of Soil CPMI with Yield, Quality, and Cumulative Nitrogen Use Efficiency in Wheat and Soil Fertility

**Figure 4:**
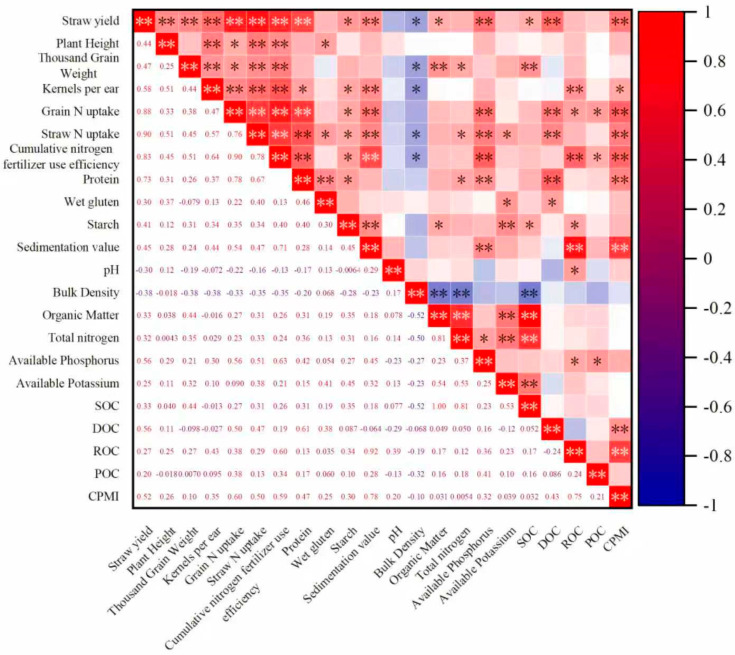
Correlation analysis of wheat yield and composition, cumulative nitrogen fertilizer use efficiency, quality, soil physical and chemical properties, and soil organic carbon and carbon pool index. Note: * indicates *p* < 0.05, and ** indicates *p* < 0.01.

Correlation analysis (Figure 4) showed that the soil CPMI was significantly positively correlated with wheat yield, quality, and nitrogen use efficiency. Specifically, soil CPMI had a highly significant (*p* < 0.01) positive correlation with wheat yield, grain and straw nitrogen uptake, cumulative nitrogen use efficiency, protein content, grain sedimentation value, and soil DOC and ROC. Cumulative nitrogen use efficiency was highly significantly (*p* < 0.01) positively correlated with yield, major agronomic traits (plant height, 1000-grain weight, and number of grains per ear), quality indicators (protein content and sedimentation value), and soil available phosphorus and ROC. Soil SOC was highly significantly (*p* < 0.01) positively correlated with 1000-grain weight, soil organic matter, total nitrogen, and available potassium and highly significantly negatively correlated with the soil bulk density. In summary, substituting chemical nitrogen fertilizer with biogas slurry increases soil organic carbon and its active portion and effectively regulates soil CPMI. This is a key approach for simultaneously improving wheat yield, quality, and cumulative nitrogen use efficiency and enhancing soil fertility.

## 3. Discussion

### 3.1. Effect of Substituting Chemical Nitrogen Fertilizer with Biogas Slurry on Wheat Yield, Quality, and Cumulative Nitrogen Use Efficiency

Proper biogas slurry application can improve wheat yield through nutrient complementation and yield component optimization. Substituting 38% of chemical fertilizer with biogas slurry can increase grain crop yield by 15–20% [30]. In our experiment, substitution treatments resulted in a significantly higher yield compared to CN (*p* < 0.05), with ONL30% treatment having an optimal effect. Moreover, the yield increase was enhanced by the extension of biogas slurry application. This is mainly attributed to the fact that biogas slurry contains large amounts of macro-elements, such as nitrogen, phosphorus, and potassium, secondary and microelements, organic carbon, and beneficial microorganisms. These components enhance the soil organic carbon content, microbial activity, and related enzyme activities, promoting crop growth [31]. Specifically, ONL30% showed a significant 8.96% increase in 1000-grain weight compared to CN in 2024. However, an excessively high proportion of biogas slurry, such as that in ONL50%, can increase the plant height, reduce lodging resistance, and result in a higher risk of yield loss. Sasada et al. [32] found that an appropriate combination of biogas slurry and chemical fertilizer improved soil physicochemical properties and microbial communities, thereby increasing crop yield.

Reasonable fertilization is essential for ensuring a high and stable crop yield. The appropriate substitution of chemical fertilizers with livestock and poultry waste, such as biogas slurry, can achieve nutrient balance in farmland ecosystems and improve crop quality [33,34,35,36]. In our study, ONL30% showed the highest starch content in two consecutive years (66.4% and 66.2%), and the grain sedimentation value increased by 13.47–20.21% compared to CN, which is consistent with the findings of Galavi et al. [37]. Additionally, the heavy metal element content in wheat grains remained below the GB 2762-2022 limit in all treatment groups, mainly because heavy metal elements tend to accumulate in biogas dregs, and their levels are relatively low in biogas slurry [38]. Substituting chemical fertilizer with biogas slurry also significantly increased the cumulative nitrogen use efficiency in wheat, with increases of 66.17–80.26% and 16.5–26.4% compared to CN and ON, respectively. Previous studies have shown that substituting chemical fertilizer with organic fertilizer can increase the cumulative nitrogen use efficiency [39,40], which aligns with the results of our study. Long-term sole application of chemical fertilizer can cause soil acidification and compaction [41,42], and sole application of biogas slurry may risk an insufficient nutrient supply at the early application stage [43]. Therefore, substituting chemical fertilizer with an appropriate proportion of biogas slurry (e.g., 30%) synergistically improves crop yield, yield stability, fertilizer use efficiency, and soil fertility [44,45,46], promoting the efficient use of agricultural resources and crop–livestock integration.

### 3.2. Effects of Substituting Chemical Fertilizer with Biogas Slurry on the Soil Physicochemical Properties and Heavy Metal Elements

As a type of manure that provides both slow-release and readily available nutrients, a biogas slurry offers long-term fertilizing effects, improving soil fertility and promoting crop growth. In this study, applying biogas slurry increased the soil nutrient content, alleviated soil acidification, and reduced the soil bulk density (Table 3). Over the two-year period, the soil pH gradually increased with the increase in the rate of biogas slurry application. Among the treatment groups, ONLY50% showed an increase of 0.40 units in soil pH over the 2 years. Farmland soil acidification has recently become a challenging issue in agriculture, and returning biogas slurry to the field is an approach for alleviating soil acidification [40]. As a liquid organic resource, biogas slurry contains nutrients that are mostly readily usable [47]. In our study, the available potassium in the soil under ONL30% was 5.59% higher than that under CN. In 2024, the available phosphorus content under ONL30% was 9.98% higher compared to that under CN. Other studies have also shown that the application of organic fertilizers, such as biogas slurry, improves the soil nutrient content. Cui et al. [48] found that proper manure application significantly increased the soil organic matter content and enhanced the biodiversity of soil microorganisms. Through a long-term field experiment, Yang et al. [49] confirmed that the combined application of manure and chemical fertilizers significantly increased the total nitrogen content in the soil, and as the proportion of manure input increased, soil total nitrogen showed an increasing trend. Hartl et al. [50] reported that after 5 consecutive years of manure application, the available potassium content of soil increased by an average of 26% compared to conventional fertilization. In addition, the use of biogas slurry promotes soil aggregate structure formation and improves soil water retention and aeration. Compared to traditional chemical fertilizers, manure enhances the soil microbial diversity [51,52]. Biogas slurry application increases soil microbial biomass and the activity of enzymes involved in the carbon and nitrogen cycles, which may increase the total soil nitrogen content [53].

Although the biogas slurry may contain pollutants, such as heavy metal elements and antibiotics, in our study, the heavy metal element content in soils under all treatments did not exceed the standard risk screening values specified in the Soil Environmental Quality—Risk Control Standard for Soil Contamination of Agricultural Land (Trial) (GB 15618-2018), indicating that biogas slurry does not cause soil heavy metal element pollution under reasonable application conditions [47,54]. However, the potential environmental and health risks of antibiotics in biogas slurry have not yet been evaluated and require further in-depth research.

### 3.3. Effects of Substituting Chemical Nitrogen Fertilizer with Biogas Slurry on the Contents of Soil Organic Carbon and Its Active Portion and Carbon Pool Management Properties

The soil organic carbon content is influenced by multiple factors, including external organic matter input, crop biomass, root exudates, and microbial activity. In our study, substituting chemical nitrogen fertilizer with biogas slurry increased the content of organic carbon and its active portion in Lime concretion black soil and improved the soil CPMI, thereby improving yield, quality, and cumulative nitrogen use efficiency in wheat and soil fertility and achieving crop–livestock integration. Specifically, the annual average increase in soil DOC in all substitution groups reached 11.93–22.93%, which is consistent with the findings of Tian et al. [55]. This is mainly because biogas slurry provides an effective carbon source for soil microorganisms, enhancing their activity, which activates soil organic carbon and promotes the accumulation of DOC, ROC, and POC. In addition, biogas slurry application enhanced the carbon sequestration capacity of topsoil, aligning with the results previously reported by other authors [56,57]. Active soil organic carbon is directly related to crop growth and soil fertility. When short-term fluctuations in total SOC are not significant, active organic carbon and CPMI serve as important indicators for evaluating soil quality and management levels [26]. In our study, substituting chemical nitrogen fertilizer with biogas slurry significantly increased the soil CPMI due to the rich organic nutrients and relatively slow mineralization rate of the biogas slurry, which effectively replenishes the depletion of soil organic matter [58], with a 30% substitution ratio showing the optimal effect.

In summary, in the field with Lime concretion black soil for wheat cultivation, partial substitution of chemical nitrogen fertilizer with biogas slurry effectively improved soil fertility and increased the soil CPMI and wheat yield and quality, thereby gaining more productivity with less cost. This provides a theoretical basis for reasonable biogas slurry application in this region. Consecutive field trials showed that the soil CPMI had a highly significant positive correlation with wheat yield, grain protein content, grain sedimentation value, and cumulative nitrogen use efficiency and soil DOC and ROC (*p* < 0.01), confirming that the improvement in the soil carbon pool is closely related to the active organic carbon portion. However, the long-term ecological effects of applying biogas slurry to farmland still need to be further clarified.

## 4. Materials and Methods

### 4.1. Experimental Field

The experiment was conducted in the field (116°18′88″ E, 33°24′98″ N) located in Weizhuang Village, Xiyang Town, Guoyang County, Bozhou City, Anhui Province. This region has a warm temperate semi-humid monsoon climate, with an average annual sunshine duration of 2015.7 h, an average temperature of 15.1 °C, annual precipitation of 851.6 mm, a frost-free period of 218 d, an elevation of approximately 25 m, and a flat terrain.

The experimental field has Lime concretion black soil, which is a semi-hydromorphic soil type with river-lake sediment as its parent material. The basic physicochemical properties of the plow layer (0–20 cm depth) are as follows: pH, 5.57; organic matter, 20.96 g∙kg^−1^; total nitrogen, 1.33 g∙kg^−1^; hydrolyzable nitrogen, 106.00 mg∙kg^−1^; available phosphorus, 42.5 mg∙kg^−1^; available potassium, 86.00 mg∙kg^−1^; and bulk density, 1.02 g∙cm^−3^. The heavy metal element contents are as follows: copper (Cu), 14.9 mg∙kg^−1^; zinc (Zn), 44.0 mg∙kg^−1^; cadmium (Cd), 0.07 mg∙kg^−1^; arsenic (As), 5.22 mg∙kg^−1^; mercury (Hg), 0.070 mg∙kg^−1^; and chromium (Cr), 33.2 mg∙kg^−1^.

### 4.2. Experimental Design

**Figure 5:**
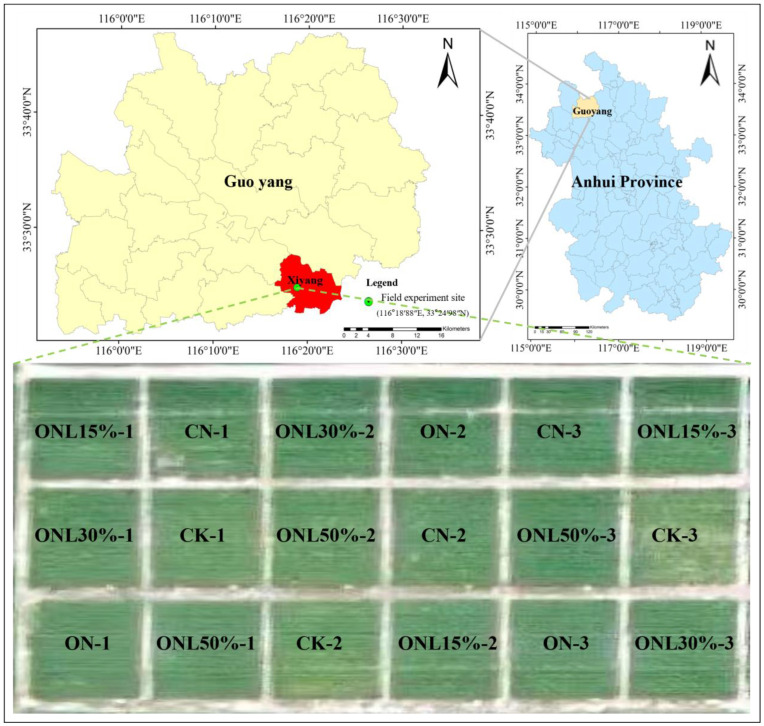
Selection of field test points and delineation of each test treatment zone.

The experiment included 6 treatments: blank control (CK, no fertilization); conventional fertilization (CN); optimized fertilization (ON); and biogas slurry substitution for chemical nitrogen fertilizer with a substitution ratio of 15% (ONL15%), 30% (ONL30%), and 50% (ONL50%). There were 3 replicates for each treatment. A completely randomized block design was adopted, with 30 m^2^ (5 m × 6 m) area for each plot. Phosphorus and potassium fertilizers were applied once as base fertilizers. Nitrogen fertilizer was applied at a base to topdressing ratio of 6:4, with the topdressing applied quantitatively through pipelines. The experiment was set up in October 2021. Fertilization management for all treatments remained the same during the wheat-growing seasons in 2023 and 2024. The biogas slurry used in the experiment was provided by the Tianpeng Family Pig Farm located in Xiyang Twon, Guoyang County, and it was produced by fermenting pig manure and urine using a static tank process. The fertilizer application rates are listed in Table 6, and the field plot arrangement is shown in Figure 5.

In 2023, the nutrient content of the biogas slurry used in the experiment were as follows: nitrogen (N), 0.03%; P, 0.0043%; K, 0.0166%; organic matter, 0.34%; pH 7.4, water, 98.78%; Hg, 0.01 mg·kg^−1^; Cu, 0.11 mg·kg^−1^; Zn, 0.20 mg·kg^−1^; As, 0.018 mg·kg^−1^; and Cd, 0.001 mg·kg^−1^. In 2024, the nutrient contents of the biogas slurry were as follows: N, 0.02%; P, 0.0086%; K, 0.0249%; organic matter, 0.28%; pH 7.3; water, 98.48%; Hg, 0.01 mg·kg^−1^; Cu, 0.10 mg·kg^−1^; Zn, 0.18 mg·kg^−1^; As, 0.015 mg·kg^−1^; and Cd, 0.001 mg·kg^−1^. The chemical fertilizers used were urea (N content, 46%), calcium superphosphate (P content, 5.16%), and potassium sulfate (K content, 41.50%).

Wheat variety ‘Guomai 9’, which is the main variety grown in local wheat production and has strong tillering and lodging and drought, was used in the study. Other conventional field management practices, such as pest control, followed those used in local wheat production. The timing of base fertilizer application, sowing, topdressing, and harvesting is given in Table 7.

### 4.3. Determinations and Methods

#### 4.3.1. Sampling and Determinations

Straw and grain yield were determined at the wheat maturity stage. Plant samples from each plot were collected from five different locations using the double diagonal method to measure the plant height, 1000-grain weight, and number of grains per ear. After threshing, the plant samples were pulverized, and the nutrient contents of straw and grains were determined. Before and after wheat harvest, fresh soil samples of about 3 kg were collected from the 0–20 cm soil layer in each plot using a soil auger following the 5-point sampling method. After thorough mixing, half of the soil samples were refrigerated at 4 °C, and a portion was air-dried and ground for measuring. The determinations and methods for both plant and soil samples are given in Table 8 [59].

#### 4.3.2. Calculation of Indicators

Using CK as the control group and the ROC content as the active organic carbon, the cumulative nitrogen use efficiency and soil CPMI were calculated based on following equations [60,61]:

(1) $$\begin{array}{c} N_{\mathrm{uptake}}({\mathrm{kg}\cdot ha}^{-1})=(Y_{g}\times N_{g})+(Y_{s}\times N_{s}) \end{array}$$

(2) $$\begin{array}{c} \mathrm{CNUE}(\%)=\frac{\left(\mathrm{NUG}+\mathrm{NUS})-({\mathrm{NUG}}_{0}+{\mathrm{NUS}}_{0}\right)}{\mathrm{TNA}}\times 100 \end{array}$$

(3) $$\begin{array}{c} \mathrm{CPMI}=\left(\frac{{\mathrm{SOC}}_{a}}{{\mathrm{SOC}}_{0}}\right)\times \left[\frac{{\mathrm{ROC}}_{a}\times \left({\mathrm{SOC}}_{0}-{\mathrm{ROC}}_{0}\right)}{\left({\mathrm{SOC}}_{a}-{\mathrm{ROC}}_{a}\right)\times {\mathrm{ROC}}_{0}}\right]\times 100 \end{array}$$

where N_uptake_ denotes plant total nitrogen uptake (kg·ha^−1^), Y_g_ grain yield (kg·ha^−1^), N_g_ grain nitrogen content (%), Y_s_ straw yield (kg·ha^−1^), and N_s_ straw nitrogen content (%). CNUE refers to the cumulative nitrogen use efficiency. NUG and NUS represent wheat grain and straw nitrogen uptake (kg·ha^−1^) of the fertilization treatment groups, respectively, and NUG_0_ and NUS_0_ are their counterparts in the control group (CK). TNA refers to the total nitrogen applied (kg·ha^−1^), and CPMI refers to the soil carbon pool management index. SOC_a_ and ROC_a_ represent the total organic carbon and active organic carbon contents (g·kg^−1^) of the soil samples in the fertilized groups, respectively, and SOC_0_ and ROC_0_ are their counterparts in the control group (CK).

### 4.4. Data Analysis

Microsoft Excel 2019 (Microsoft, Redmond, WA, USA) and IBM SPSS Statistics 26.0 (IBM, Armonk, NY, USA) were used for data processing and variance analysis. Graphs were plotted using OriginPro 2021 (OriginLab, Northampton, MA, USA). One-way analysis of variance (ANOVA) was applied to analyze the effects of different fertilization treatments on the soil carbon pool, soil fertility, and yield, quality, and cumulative nitrogen use efficiency of wheat, with significance considered at *p* < 0.05.

## 5. Conclusions

Proper biogas slurry application may optimize wheat yield components, thereby improving grain yield and quality indicators, such as the starch, wet gluten, and protein contents. In this study, wheat was cultivated in a field with Lime concretion black soil, and the substitution of 30% chemical nitrogen fertilizer with biogas slurry resulted in the highest wheat yield (9.06 t·ha^−1^) and optimal quality. By increasing the soil organic matter content and regulating carbon pool properties, biogas slurry application increased the soil pH, reduced the soil bulk density, effectively improved the physicochemical properties of Lime concretion black soil, and significantly increased the cumulative nitrogen use efficiency of wheat. Applying biogas slurry in the wheat field with Lime concretion black soil in the Northern Anhui Plain of China may increase soil CPMI, enhance soil fertility, increase cumulative nitrogen use efficiency, and improve wheat yield and quality.

## Figures and Tables

**Table 1:** Effects of different fertilization treatments on wheat yield components.

Year	Treatment	Plant Height (cm)	Thousand Grain Weight (g)	Kernels Per Ear
2023	CK	71.21 ± 3.34 b	39.44 ± 0.82 b	36.33 ± 2.65 c
	CN	72.24 ± 3.75 b	40.50 ± 1.69 b	43.44 ± 3.91 ab
	ON	70.71 ± 0.91 b	43.30 ± 0.76 a	43.56 ± 0.69 ab
	ONL15%	72.86 ± 3.53 b	40.49 ± 0.77 b	46.44 ± 0.84 a
	ONL30%	66.66 ± 3.68 b	40.20 ± 1.09 b	39.22 ± 3.34 bc
	ONL50%	79.32 ± 3.45 a	41.02 ± 1.10 b	44.89 ± 3.86 a
2024	CK	63.61 ± 0.35 c	39.09 ± 0.56 d	35.78 ± 3.10 b
	CN	71.06 ± 0.79 b	40.03 ± 0.51 cd	41.33 ± 2.33 ab
	ON	70.22 ± 0.92 b	42.69 ± 1.24 ab	43.67 ± 5.17 ab
	ONL15%	74.5 ± 3.24 b	40.81 ± 0.25 c	47.78 ± 2.04 a
	ONL30%	72.61 ± 3.25 b	43.62 ± 1.18 a	46.56 ± 1.95 a
	ONL50%	79.11 ± 1.9 a	41.38 ± 0.01 bc	46.78 ± 0.84 a

Notes: Mean ± standard deviation. Values followed by different letters within the column are significantly different at *p* < 0.05.

**Table 2:** Effects of different fertilization treatments on heavy metal content in wheat grains.

Year	Treatment	Pb (mg·kg^−1^)	Cd (mg·kg^−1^)	Hg (mg·kg^−1^)	As (mg·kg^−1^)	Ni (mg·kg^−1^)	Cr (mg·kg^−1^)	Cu (mg·kg^−1^)	Zn (mg·kg^−1^)
2023	CK	ND	0.04 ± 0.00 a	ND	0.01 ± 0.01 a	ND	0.01 ± 0.02 a	3.56 ± 0.34 a	21.90 ± 3.29 c
	CN	ND	0.04 ± 0.01 a	ND	0.01 ± 0.01 a	ND	ND	3.76 ± 0.56 a	30.00 ± 2.36 ab
	ON	ND	0.04 ± 0.00 a	ND	0.00 ± 0.01 a	ND	0.01 ± 0.02 a	4.18 ± 0.28 a	31.67 ± 1.70 a
	ONL15%	ND	0.04 ± 0.00 a	ND	0.01 ± 0.01 a	ND	ND	3.85 ± 0.23 a	28.20 ± 2.29 ab
	ONL30%	ND	0.05 ± 0.00 a	ND	0.01 ± 0.01 a	ND	0.06 ± 0.07 a	3.69 ± 0.24 a	26.37 ± 2.00 bc
	ONL50%	ND	0.05 ± 0.00 a	ND	0.01 ± 0.01 a	ND	ND	3.75 ± 0.54 a	27.20 ± 3.84 ab
2024	CK	ND	0.04 ± 0.01 a	ND	0.00 ± 0.01 c	ND	0.33 ± 0.08 a	3.30 ± 0.10 d	21.90 ± 3.29 d
	CN	ND	0.04 ± 0.01 a	ND	0.02 ± 0.01 ab	ND	ND	3.80 ± 0.10 c	31.50 ± 0.44 c
	ON	ND	0.04 ± 0.00 a	ND	0.01 ± 0.00 bc	ND	0.31 ± 0.06 a	3.73 ± 0.06 c	30.17 ± 3.01 bc
	ONL15%	0.06 ± 0.02 b	0.05 ± 0.01 a	0.01 ± 0.01 a	0.02 ± 0.01 ab	ND	ND	4.10 ± 0.10 b	28.20 ± 2.29 ab
	ONL30%	0.07 ± 0.00 b	0.05 ± 0.00 a	0.01 ± 0.01 a	0.02 ± 0.01 ab	ND	0.35 ± 0.10 a	4.14 ± 0.05 b	26.37 ± 2.00 a
	ONL50%	0.10 ± 0.00 a	0.05 ± 0.00 a	0.01 ± 0.00 a	0.03 ± 0.01 a	ND	ND	4.51 ± 0.29 a	27.20 ± 3.84 ab
Limit value (GB 2762-2022)		0.20	0.10	0.02	0.50	1.00	1.0	/	/

Notes: Mean ± standard deviation. “ND” represents data not detected. Values followed by different letters within the column are significantly different at *p* < 0.05.

**Table 3:** Effects of different fertilization treatments on soil physical and chemical properties of wheat.

Year	Treatment	pH	Bulk Density (g·cm^−3^)	Total Nitrogen (g·kg^−1^)	Available Phosphorus (mg·kg^−1^)	Available Potassium (mg·kg^−1^)
2023	CK	6.11 ± 0.42 a	1.21 ± 0.07 a	1.42 ± 0.18 a	24.47 ± 2.55 a	261.00 ± 55.49 a
	CN	5.63 ± 0.09 ab	1.20 ± 0.08 a	1.39 ± 0.16 a	40.27 ± 21.04 a	290.67 ± 101.04 a
	ON	5.59 ± 0.12 b	1.07 ± 0.07 a	1.48 ± 0.14 a	36.83 ± 13.05 a	296.33 ± 55.05 a
	ONL15%	5.65 ± 0.27 ab	1.17 ± 0.12 a	1.38 ± 0.18 a	44.07 ± 10.66 a	261.33 ± 23.03 a
	ONL30%	5.88 ± 0.41 ab	1.14 ± 0.13 a	1.44 ± 0.14 a	42.27 ± 11.54 a	295.67 ± 61.98 a
	ONL50%	5.79 ± 0.36 ab	1.18 ± 0.11 a	1.36 ± 0.04 a	43.50 ± 22.13 a	273.00 ± 75.36 a
2024	CK	6.09 ± 0.10 a	1.22 ± 0.04 a	1.33 ± 0.05 c	24.50 ± 0.10 c	250.67 ± 6.42 c
	CN	5.61 ± 0.23 b	1.22 ± 0.01 a	1.37 ± 0.04 bc	39.36 ± 0.57 b	289.47 ± 10.44 b
	ON	5.58 ± 0.12 b	1.15 ± 0.06 ab	1.41 ± 0.01 b	35.76 ± 0.94 b	286.75 ± 9.80 b
	ONL15%	6.07 ± 0.07 a	1.14 ± 0.06 ab	1.41 ± 0.02 b	44.34 ± 2.89 a	296.93 ± 4.82 b
	ONL30%	6.10 ± 0.18 a	1.13 ± 0.07 ab	1.49 ± 0.02 a	43.29 ± 3.14 a	316.87 ± 1.62 a
	ONL50%	6.19 ± 0.03 a	1.11 ± 0.02 b	1.42 ± 0.06 ab	45.12 ± 2.80 a	318.40 ± 5.54 a

Notes: Mean ± standard deviation. Values followed by different letters within the column are significantly different at *p* < 0.05.

**Table 4:** Effects of different fertilization treatments on soil heavy metal content.

Year	Treatment	Cd (mg·kg^−1^)	Hg (mg·kg^−1^)	As (mg·kg^−1^)	Pb (mg·kg^−1^)	Cr (mg·kg^−1^)	Cu (mg·kg^−1^)	Ni (mg·kg^−1^)	Zn (mg·kg^−1^)
2023	CK	0.10 ± 0.00 a	0.07 ± 0.01 b	5.92 ± 1.32 a	23.00 ± 2.00 a	33.67 ± 1.53 a	16.67 ± 1.53 a	19.33 ± 1.15 c	47.00 ± 2.65 a
	CN	0.09 ± 0.01 a	0.07 ± 0.00 b	6.69 ± 0.86 a	23.00 ± 3.00 a	35.00 ± 6.24 a	15.67 ± 1.53 a	20.33 ± 3.06 ab	48.33 ± 1.15 a
	ON	0.09 ± 0.01 a	0.07 ± 0.00 b	6.17 ± 0.08 a	20.00 ± 2.65 a	39.00 ± 4.36 a	15.67 ± 0.58 a	19.33 ± 0.58 ab	46.67 ± 0.58 a
	ONL15%	0.10 ± 0.02a	0.11 ± 0.05 a	5.72 ± 0.89 a	20.00 ± 2.00 a	40.00 ± 2.00 a	15.67 ± 0.58 a	17.33 ± 1.15 b	47.33 ± 2.08 a
	ONL30%	0.10 ± 0.01 a	0.08 ± 0.00 b	5.81 ± 0.91 a	22.33 ± 2.08 a	40.00 ± 2.00 a	17.00 ± 1.00 a	20.00 ± 1.00 ab	50.00 ± 1.73 a
	ONL50%	0.09 ± 0.01 a	0.07 ± 0.01 b	6.02 ± 0.58 a	21.67 ± 3.06 a	34.67 ± 7.09 a	15.33 ± 0.58 a	18.67 ± 0.58 ab	49.00 ± 2.65 a
2024	CK	0.04 ± 0.01 b	0.09 ± 0.07 b	7.50 ± 0.47 d	19.67 ± 1.53 b	35.00 ± 3.61 b	15.34 ± 0.42 c	24.00 ± 1.00 c	57.00 ± 6.24 b
	CN	0.04 ± 0.00 bc	0.10 ± 0.07 b	7.62 ± 0.55 c	17.67 ± 0.58 ab	39.33 ± 4.16 ab	15.23 ± 0.54 ab	21.67 ± 2.08 ab	55.00 ± 2.65 b
	ON	0.04 ± 0.01 c	0.05 ± 0.00 b	7.75 ± 0.43 cd	18.33 ± 0.58 ab	41.33 ± 4.04 b	14.83 ± 1.11 bc	22.00 ± 1.73 bc	58.00 ± 4.36 b
	ONL15%	0.04 ± 0.01 a	0.05 ± 0.01 a	7.51 ± 0.28 b	17.67 ± 2.08 ab	38.67 ± 4.93 a	13.95 ± 1.02 abc	22.67 ± 2.08 ab	62.33 ± 2.52 a
	ONL30%	0.04 ± 0.00 a	0.05 ± 0.01 a	7.74 ± 0.49 a	20.67 ± 1.53 ab	39.00 ± 5.20 a	15.05 ± 0.64 a	22.67 ± 0.58 a	59.67 ± 2.31 a
	ONL50%	0.05 ± 0.01 a	0.05 ± 0.01 a	7.75 ± 0.67 a	20.33 ± 2.08 a	40.00 ± 5.29 a	14.83 ± 1.53 ab	22.00 ± 1.00 a	57.00 ± 4.58

Notes: Mean ± standard deviation. Values followed by different letters within the column are significantly different at *p* < 0.05.

**Table 5:** Effects of different fertilization treatments on soil organic carbon and its active components.

Year	Treatment	SOC (g·kg^−1^)	DOC (mg·kg^−1^)	ROC (g·kg^−1^)	POC (g·kg^−1^)	CPMI
2023	CK	14.42 ± 1.29 a	28.27 ± 0.52 d	3.36 ± 0.13 c	4.23 ± 1.06 a	98.34 ± 7.63 c
	CN	13.73 ± 0.92 a	32.50 ± 2.70 c	3.50 ± 0.09 c	4.42 ± 2.43 a	105.10 ± 2.23 c
	ON	14.68 ± 0.64 a	31.62 ± 0.59 cd	3.46 ± 0.07 c	4.38 ± 0.60 a	101.46 ± 2.01 c
	ONL15%	13.13 ± 0.91 a	35.22 ± 2.99 bc	4.02 ± 0.07 a	5.00 ± 2.74 a	129.55 ± 1.38 b
	ONL30%	14.66 ± 1.04 a	38.02 ± 2.54 ab	4.56 ± 0.13 a	5.08 ± 1.16 a	148.66 ± 8.95 a
	ONL50%	13.71 ± 0.60 a	41.29 ± 1.56 a	4.23 ± 0.25 a	4.41 ± 1.41 a	137.62 ± 14.36 b
2024	CK	12.93 ± 0.03 c	11.43 ± 0.82 c	3.87 ± 0.17 c	3.91 ± 0.12 d	99.28 ± 4.76 d
	CN	14.42 ± 0.75 ab	12.33 ± 0.59 c	3.91 ± 0.07 c	4.30 ± 0.32 cd	96.25 ± 1.95 d
	ON	14.42 ± 0.75 ab	14.43 ± 0.40 ab	3.89 ± 0.06 c	4.62 ± 0.23 bc	93.59 ± 1.00 d
	ONL15%	13.94 ± 0.90 ab	14.96 ± 0.62 a	4.52 ± 0.19 b	5.38 ± 0.39 a	117.62 ± 3.83 c
	ONL30%	14.83 ± 0.20 ab	15.45 ± 0.46 a	5.11 ± 0.10 a	4.85 ± 0.29 b	137.08 ± 4.87 a
	ONL50%	14.20 ± 0.39 a	13.81 ± 0.33 b	4.72 ± 0.07 b	4.89 ± 0.06 b	124.49 ± 4.15 b

Notes: Mean ± standard deviation. Values followed by different letters within the column are significantly different at *p* < 0.05.

**Table 6:** Fertilizer quantities for different fertilization treatments in 2023 and 2024 wheat seasons.

Year	Treatment	Organic Fertilizer (kg·ha^−1^)	Fertilizer (kg·ha^−1^)			Converted to Pure Nutrients (kg·ha^−1^)		
		Biogas Slurry	Urea	Superphosphate	Potassium Chloride	N	P	K
2023	CK	0.00	0.00	0.00	0.00	0.00	0.00	0.00
	CN	0.00	284.63	238.76	120.06	220.00	45.15	49.80
	ON	0.00	258.75	227.39	120.06	200.00	43.00	49.80
	ONL15%	91.23	194.06	209.82	91.69	200.00	43.00	49.80
	ONL30%	182.46	129.38	192.25	63.33	200.00	43.00	49.80
	ONL50%	304.10	43.12	168.82	25.51	200.00	43.00	49.80
2024	CK	0.00	0.00	0.00	0.00	0.00	0.00	0.00
	CN	0.00	284.63	238.76	120.06	220.00	45.15	49.80
	ON	0.00	258.75	227.39	120.06	200.00	43.00	49.80
	ONL15%	157.10	194.06	139.13	27.23	200.00	43.00	49.80
	ONL30%	314.21	129.38	50.87	0.00	200.00	43.00	49.80
	ONL50%	523.68	43.12	0.00	0.00	200.00	43.00	49.80

**Table 7:** Field management dates in 2023 and 2024 wheat seasons.

Management Measure	2023	2024
Base fertilizer and sowing	27 October 2022	24 October 2023
Application of fertilizer and urea	8 March 2023	15 March 2024
Harvest	8 June 2023	31 May 2024

**Table 8:** Indicators and methods of plant and soil measurement.

Sample	Measurement Indexes	Measurement Methods
Plant	Settlement index	GB/T 15685-2011 Grain Oil Inspection Determination of Wheat Sedimentation Index by SDS Method
	Starch	GB 5009.9-2016 National Food Safety Standard Determination of Starch in Food
	Wet gluten	GB/T 5506.1-2008 Wheat and Wheat Flour Gluten Content Part 1: Wet Gluten Determination by Hand Washing
	Protein	GB 5009.5-2016 National Food Safety Standard Determination of Protein in Food
Soil	pH	DMP-2 mv/pH Potentiometry, extracted with water
	Unit weight	Standard ring knife method
	Organic matter	Potassium dichromate oil bath plus heat capacity method
	TN	Kjeldahl method for nitrogen determination
	Available P	Extraction with NaHCO_3_ and determination by spectrophotometry
	Quick-acting potassium	Flame photometry Extract with ammonium acetate, and determination by resistance spectrophotometry (Sherwood M410/M420, Nanodrop, Germany)
	Cation exchange capacity	Ammonium acetate exchange method
	SOC	Potassium dichromate capacity method-external heating method
	DOC	Total organic carbon (TOC) instrument (vario TOC Cube, Elementar, Hanau, Germany) determination
	ROC	333 mmol/L potassium permanganate colorimetric method
	POC	Cambardella

## Data Availability

The original contributions presented in this study are included in the article. Further inquiries can be directed to the corresponding author.
